# *Myroides odoratimimus* Forms Structurally Complex and Inherently Antibiotic-Resistant Biofilm in a Wound-Like *in vitro* Model

**DOI:** 10.3389/fmicb.2017.02591

**Published:** 2017-12-22

**Authors:** Arianna Pompilio, Giuseppe Galardi, Fabio Verginelli, Maurizio Muzzi, Andrea Di Giulio, Giovanni Di Bonaventura

**Affiliations:** ^1^Department of Medical, Oral & Biotechnological Sciences, “G. d'Annunzio” University of Chieti-Pescara, Chieti, Italy; ^2^Aging Research Center and Translational Medicine, “G. d'Annunzio” University of Chieti-Pescara, Chieti, Italy; ^3^Department of Pharmacy, “G. d'Annunzio” University of Chieti-Pescara, Chieti, Italy; ^4^Department of Science, LIME, University of Roma Tre, Rome, Italy

**Keywords:** *Myroides odoratimimus*, antibiotic-resistance, biofilm, wound-like *in vitro* model, recurrent ulcer infection

## Abstract

*Myroides odoratimimus* is an aerobic, non-fermenting Gram-negative multidrug-resistant bacterium widely distributed in nature that rarely causes infections in immunocompromised patients. We recently described in a diabetic patient a case of recurrent calcaneal ulcer infection caused by a *M. odoratimimus* strain showing potential for biofilm formation. For the first time, we therefore evaluated the ability of *M. odoratimimus* to form biofilm under different pH values and glucose concentrations using an *in vitro* “skin-like” model, and its susceptibility to levofloxacin, meropenem, and tigecycline. The expression of some antibiotic-resistance related genes was also monitored by RT-PCR during planktonic-to-biofilm transition. Our results indicated that *M. odoratimimus* can produce relevant amounts of biofilm biomass, in a time-dependent manner, especially at acidic pH and regardless of glucose concentration tested. The comparative analysis of MIC and MBC values between planktonic and sessile cells showed that resistance to antibiotics increased during the planktonic-to-biofilm transition. Viable cell count indicated that none of the tested antibiotics were able to completely eradicate preformed biofilms, although meropenem and levofloxacin were the most active causing a significant, dose-independent, reduction of biofilm's viability, as also confirmed by microscopic analysis. RT-PCR showed that antibiotic-resistance related *gyrA* and *acrB* genes are over-expressed during the transition from planktonic to sessile (biofilm) lifestyle. Overall, our findings showed that *M. odoratimimus* can form relevant amounts of inherently antibiotic-resistant biofilm under conditions relevant to wound site, therefore suggesting a role in the pathogenesis of chronic ulcer infections.

## Introduction

*Myroides odoratimimus* is a non-motile, non-fermentative, strictly aerobic, Gram-negative rod with a characteristic fruity odor and yellow pigmentation due to the flexirubin pigment (Vancanneyt et al., 1996). Considered as a low-grade opportunistic pathogen, *M. odoratimimus* is rarely isolated in the clinical setting causing life-threatening infections, mainly in the immunocompromised host (Benedetti et al., 2011).

*Myroides odoratimimus* is widely distributed in the environment, especially in water. To date, only eight cases of *M. odoratimimus* infection have been reported in the literature. Generally acquired as consequence of contact with contaminated water, infections are isolated cases of pneumonia, endocarditis, urinary tract infections, ventriculitis, and skin infections with septicaemia, as well as nosocomial outbreaks (MacFarlane et al., 1985; Bachmeyer et al., 2007; Douce et al., 2008; Benedetti et al., 2011; Ktari et al., 2012; Maraki et al., 2012; Crum-Cianflone et al., 2014).

Recently we described, for the first time, a case of a recurrent post-traumatic calcaneal ulcer infection caused by *M. odoratimimus*, in a diabetic male (Pompilio et al., 2017). The recurrent nature of the infection together with the evidence that *in vitro* the strain formed relevant amount of biofilm, led us to hypothesize that *M. odoratimimus* could be able to form biofilm under experimental conditions relevant to wound infections. A growing body of published works, in fact, indicated that in chronic wounds antibiotic-resistant biofilm formation contributes to a delay in healing (Percival et al., 2012; Rahim et al., 2017).

In the present work we, therefore, evaluated the biofilm forming ability of *M. odoratimimus* using an *in vitro “skin-like”* model consisting of a tridimensional network of collagen type I and “simulated wound fluid” to mimicry the serous exudate. In particular, we first evaluated kinetics of biofilm formation over 7 days at pH values (5.5, 7.2, and 8.5) relevant for chronic wounds resolution (Gethin, 2007), and in the presence of glucose at concentrations relevant to different stages of diabetic disease (5, 12.5, and 25 mM) (American Diabetes Association, 2017). The susceptibility of biofilm to bactericidal concentrations of therapeutically relevant antibiotics was then assessed both by viable cell count and confocal and electronic microscopy. Finally, changes in expression levels of three genes involved in *M. odoratimimus* antibiotic-resistance were evaluated, by real-time PCR, during the planktonic-to-biofilm transition.

## Materials and methods

### Bacterial strain and growth conditions

The bacterial strain tested in the present study was isolated from a 65-year-old male diabetic patient came to surgical examination for the clinical deterioration of a recurrent post traumatic ulcer located on the calcaneal region of the right foot (Pompilio et al., 2017). Initial identification of *M. odoratimimus* was carried out by Vitek 2 (bioMèrieux) and subsequently confirmed by MALDI-TOF with a score of 2.322 (Bruker Daltonik MALDI Biotyper, Bremen, Germany). The final identification was accomplished by 16S rDNA sequencing, revealing a 100% identity with *M. odoratimimus* strain PR63039 (accession number NZ_CP013690). The strain was stored at −80°C in Microbank system (Biolife Italiana S.r.l., Milan, Italy) until use when it was grown in trypticase soy broth (Oxoid SpA, Garbagnate M.se, Milan, Italy), then twice on Mueller-Hinton agar (MHA; Oxoid SpA) to check for absence of contaminants and to regain the original phenotypical traits.

### Identification of *M. odoratimimus* by rDNA16S sequencing

Bacterial DNA was isolated using the QIAmp DNA Mini Kit (Qiagen Inc., Chatsworth, CA, USA) and quantified with Nanodrop 2000 (Thermo Fisher Scientific, Waltham, MA, USA). rDNA 16S typing was performed via PCR as previously described (Schröttner et al., 2014).

### Standardization of bacterial inoculum

Some colonies from an overnight 37°C growth onto MHA (Oxoid SpA) were resuspended in TSB (Oxoid SpA) and incubated overnight at 37°C. The broth culture was then equilibrated with fresh TSB to an optical density measured at 550 nm (OD_550_) of 1.2 (corresponding to 1-5x10^9^ CFU/ml). This standardized bacterial suspension was then diluted (vol/vol) accordingly to use: (i) 1:100 in fresh TSB, for biofilm formation assay onto polystyrene; (ii) 1:400 in Simulated Wound Fluid (SWF) (Werthén et al., 2010) consisting of 50% Fetal Bovine Serum (FBS) (Euroclone SpA; Pero, Milan, Italy) and 50% Peptone Water (PW) (Oxoid SpA) in 0.1% sterile saline, for kinetics of biofilm formation in collagen gel matrix. The effect of pH and glucose concentration was evaluated by adjusting SWF—by using HCl 37% or NaOH 1M (both from Sigma-Aldrich, Milan, Italy)—to three different pH values (5.5, 7.2, and 8.5) and by adding D-(+)-glucose solution 45% (Sigma-Aldrich) to obtain 5, 12.5, and 25 mM final concentrations.

### Kinetics of biofilm formation on polystyrene

Two-hundred microliters of the standardized inoculum (1-5x10^7^ CFU/ml) were inoculated in each well of a 96 well, flat-bottom, polystyrene microtiter plate (Falcon BD, Corning, NY, USA), and incubated statically at 37°C. Following 24 and 96 h-incubation, microtiter plate was washed twice with 200 μl sterile PBS, fixed (60°C, 1 h) and stained (room temperature, 5 min) with 200 μL of 1% crystal violet modified according to Hucker et al. (Hucker, 1921). Wells were then washed with tap water until complete removal of unbound stain, then air-dried (37°C, 30 min). Biofilm samples were destained with 200 μl of 33% glacial acetic acid, the acetic acid solution was transferred to another microtiter plate, and finally spectrophotometrically measured (OD_492_) by SUNRISE^TM^ microplate reader (Tecan Group Ltd, Männedorf, Switzerland).

### Collagen gel matrix preparation

Collagen gel matrix was prepared according to Werthén et al. (2010), with some modifications. Briefly, 10 ml of collagen solution at 2 mg/ml were prepared in ice by mixing 1 ml acetic acid 0.1% (Sigma-Aldrich), 2 ml of bovine collagen type I 10 mg/ml (FibriCol; CellSystems Biotechnologie Vertrieb GmbH, Troisdorf, Germany), 6 ml of cold SWF (3 ml FBS + 3 ml PW), and finally 1 ml NaOH 0.1 M. Aliquots (100 μl) of this solution were poured in each well of a 48 well, tissue culture-treated, flat bottom, polystyrene microplate (Iwaki; Asahi Glass Co., Ltd., Japan), then allow to polymerize at 37°C for 1 h.

### Kinetics of biofilm formation in collagen gel matrix

Two-hundred microliters of the standardized inoculum (1–5 × 10^6^ CFU/ml) were inoculated at the surface of each collagen gel matrix prepared in 48 well microtiter plate. A control sample was exposed only to sterile SWF. Samples were then incubated at 37°C, replacing daily with 200 μl SWF. At prefixed times (1, 2, 3, 4, 5, 6, and 7 days), each sample was washed twice with 200 μl PBS, then exposed to 100 μl collagenase 1 mg/ml (Advanced BioMatrix, Carlsbad, USA) at 37°C for 1 h to solubilize gel matrix. Each sample was then collected, vortexed, and centrifuged (4,000 rpm, 10 min, 4°C). The pellet was then resuspended in 1 ml PW and underwent to viable cell count onto MHA.

### Antibiotic susceptibility of planktonic and biofilm cells

The susceptibility of both planktonic and biofilm cells to levofloxacin, meropenem, and tigecycline (all from Sigma-Aldrich) was evaluated by measuring MIC and MBC values, according to CLSI guidelines (CLSI, 2017). *Pseudomonas aeruginosa* ATCC27853 (levofloxacin, meropenem), and *Staphylococcus aureus* ATCC29213 (tigecycline) were used as control strains. Antibiotic susceptibility of biofilm was evaluated in three different ways: (i) MIC and MBC values of sessile cells obtained following disruption of 7 day-old biofilm grown at different pH values (5.5, 7.2, and 8.5) and in the presence of glucose 12.5 mM; (ii) viability of preformed gel-embedded 7 day-old biofilm (37°C, TSB with pH 7.2 and glucose 12.5 mM) was assessed by viable counts following exposure for 24 h to bactericidal concentrations of levofloxacin (64–1,024 μg/ml), meropenem (128–1,024 μg/ml), and tigecycline (4–512 μg/ml) prepared in cation-adjusted Muller-Hinton broth (CAMHB) (Oxoid SpA).

### Microscopic observation

The ultrastructure of *M. odoratimimus* biofilm was evaluated by both Confocal Laser Scanning Microscopy (CLSM) and Focused Ion Beam Scanning Electron Microscopy (FIB-SEM).

- CLSM. Biofilm samples were grown in collagen gel matrix for 1 and 7 days (37°C, TSB at pH 7.2 and glucose 12.5 mM) in each well of the chambered coverslip μ-Slide 8 well (Ibidi; Martinsried, Germany). In a parallel series of experiments, 7 day-old biofilms exposed for 24 h to tigecycline, levofloxacin or meropenem at 512 μg/ml in CAMHB, or to CAMHB only (controls), were also set. Samples were stained with Live/Dead BacLight to assess viability, and Concanavalin A (ConA; Alexa Fluor 647 Conjugate) to detect extracellular polymeric substance (EPS) (both from Thermo Fisher Scientific). CLSM analysis was performed with an LSM 510 META laser scanning microscope attached to an Axioplan II microscope (Zeiss Italia, Arese, Milan, Italy). To determine biofilm structure, a Z-series of 25 optical planes at xy resolution of 512 × 512 pixel (68.4 × 68.4 μm) with a thickness of 1.00 μm was taken throughout the biofilm. Representative images were acquired using ZEN 2.3 SP1 software (Carl Zeiss; ver. 14.0) and analyzed by COMSTAT software (ver. 2.1) to measure total biomass, substratum coverage, and maximum thickness (Heydorn et al., 2000).
- FIB/SEM. Seven-day-old biofilms grown (TSB with pH 7.2 and glucose 12.5 mM) in 48 well tissue culture-treated, flat bottom, polystyrene microtiter plate (Iwaki) were exposed for 24 h to each antibiotic at 512 μg/ml in CAMHB, or to CAMHB only (controls). Samples were washed in PBS, then fixed in a mixture of 2% [vol/vol] paraformaldehyde (Electron Microscopy Sciences, Hatfield, PA, USA) + 2% glutaraldehyde (Sigma-Aldrich) [vol/vol] in 0.15 M sodium cacodylate buffer (pH 7.4; Fluka). Samples were post-fixed for 90 min in 1% OsO_4_ (Electron Microscopy Sciences) in 0.15 M cacodylate buffer, dehydrated in ascending ethanol series, en-bloc stained with 2% alcoholic uranyl acetate for 60 min and rinsed in 100% ethanol. Samples were embedded in epoxy embedding medium (Sigma-Aldrich), using propylene oxide (Sigma-Aldrich) as a bridging solvent, and left to polymerize for 3 days at 60°C. Thick sequential slices of about 15–20 μm were cut with a glass knife on a Leica Ultracut T ultramicrotome, mounted on stubs using double-sided adhesive carbon disks and gold coated in an Emitech K550 unit. Gold-sputtered samples were analyzed with the Dualbeam FIB/SEM Helios Nanolab microscope (FEI, Hillsboro, USA). Regions containing the biofilm were located by using secondary electrons and cross-sectioned by the focused gallium ion beam operated at 30 kV and 0.92 nA. Pictures of each cross-section were acquired using backscattered electrons with an operating voltage of 2 kV and a current of 0.17 nA.

Both CLSM and FIB-SEM representative images were acquired and processed for display using Photoshop (Adobe Systems Inc., San Jose, California) software.

### Real-time PCR

The expression of gyrase subunit A (*gyrA*), β-lactamase MUS-1 (*MUS-1*), and efflux pump AcrB (*acrB*) during planktonic-to-biofilm transition was assessed by real-time PCR (RT-PCR) as previously described by Pompilio et al. (2015), with minor modifications. Briefly, RNA was isolated using TRIzol (Invitrogen) from the following samples obtained in SWF (pH 7.2, glucose 12.5 mM): (i) planktonic cells during log-phase (following 1 day-incubation, corresponding to an OD_550_ = 0.3); (ii) planktonic cells during stationary phase (following 3 day-incubation, corresponding to an OD_550_ = 0.6); (iii) 24 h-old biofilm cells; (iv) 4 day-old biofilm cells; and (v) 7 day-old biofilm cells. RT-PCR was carried out using PrimeTime Gene Expression Master Mix (IDT Inc., Coralville, Iowa, USA) and Universal Probe Library RT-PCR assays (Roche Diagnostic). The primers and probes used are listed in Table 1. Each sample was run, in triplicate, in 7900HT Real-Time PCR platform, and gene expression was normalized to that of the housekeeping gene *rpoA* according to the 2–ΔCT method.

**Table 1:** Primer sequences for RT-PCR.

**Gene**	**Primers sequence**	**UPL probe (#)^a^**
*gyrA*	FW: 5′-actctatcgcagctgtatgtaagg-3′	#53
	Rv: 5′-aatgattgtatcaacatctccttcta-3′	
*MUS-1*	FW: 5′-gataataccgtagtctggtttccaa-3′	#88
	Rv: 5′-agtggtagcttcggcactct-3′	
*acrB*	FW: 5′-agggtagctcaggtgacagg-3′	#62
	Rv: 5′-agtgattacccccgcttgta-3′	
*rpoA*^b^	FW: 5′-tcatacgattcagtttgtgcaat-3′	#34
	Rv: 5′-gctgcaaaaacattaatacacca-3′	

a *Universal Probe Library (UPL) code number*.

b *Housekeeping gene*.

### Statistical analysis

Each experiment was carried out in triplicate and repeated on at least two different occasions. Statistical analysis was conducted using GraphPad software (ver. 7.0, GraphPad Inc, San Diego USA), considering as significant *p*-values less than 0.05. The statistical significance of differences was evaluated using ANOVA following by Tukey's multiple comparison test (kinetics of biofilm formation in collagen gel matrix, activity of antibiotics against preformed biofilm) or *t*-test (kinetics of biofilm formation on polystyrene, image analysis by COMSTAT software, gene expression).

## Results

### Kinetics of biofilm formation on polystyrene

**Figure 1:**
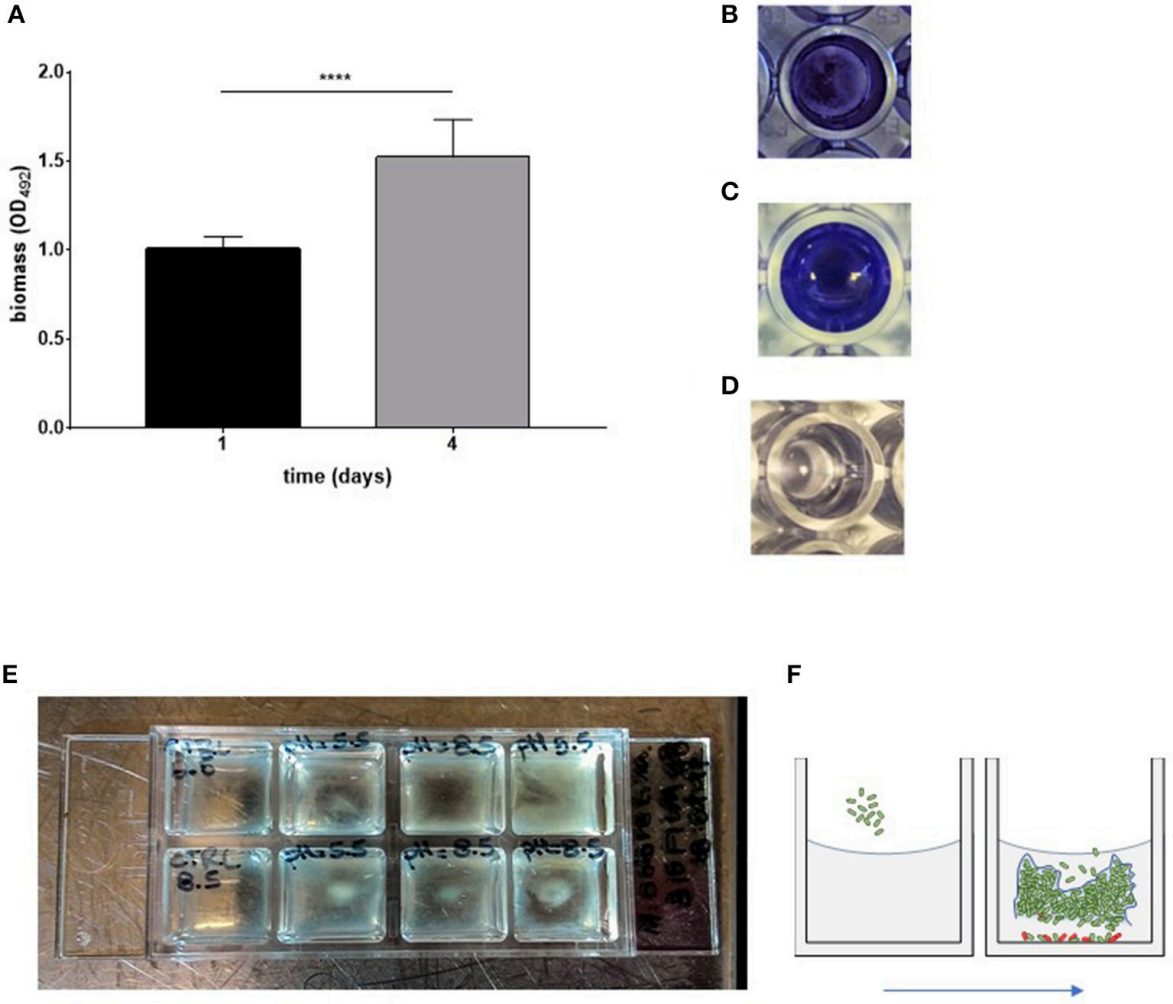
*in vitro* models to assess biofilm formation by *Myroides odoratimimus*. **(A–D)** Biofilm formation in 96-well polystyrene microtiter plate. **(A)** The biofilm biomass formed in TSB following 1 and 4 days of incubation at 37°C was assessed by crystal violet assay. Results are shown as mean optical density at 492 nm (OD_492_) + SD (*n* = 40). ^****^p < 0.0001, paired *t*-test, day 1 vs. day 4. **(B**) Biofilm sample formed in a well of a 96 well microtiter after crystal violet stain. **(C)** Biofilm sample following crystal violet extraction by glacial acetic acid. **(D)** Not inoculated (control) sample. **(E,F)** Biofilm formation in “skin-like” model (Werthén et al., 2010). **(E)** Biofilm was allowed to form in each well of a chambered coverslip μ-Slide 8-well, within three-dimensional matrix consisting of collagen type I and “simulated wound fluid” (SWF) to mimicry the serous exudate. Biofilm formation under different pH values (5.5 and 8.5) is indicated by the opacity observable within each well in the second, third and fourth column. Uninfected wells (control) are in the first column. **(F)** Schematic representation of biofilm formation within collagen gel matrix.

The ability to form biofilm on polystyrene over time was spectrophotometrically evaluated by crystal violet assay. The strain resulted in being a strong biofilm producer, according to criteria proposed by Stepanović et al. (2007), and biofilm biomass increased over time (day 1 vs. day 4; *p* < 0.0001) (Figures 1A–D).

### Kinetics of biofilm formation in collagen gel matrix

**Figure 2:**
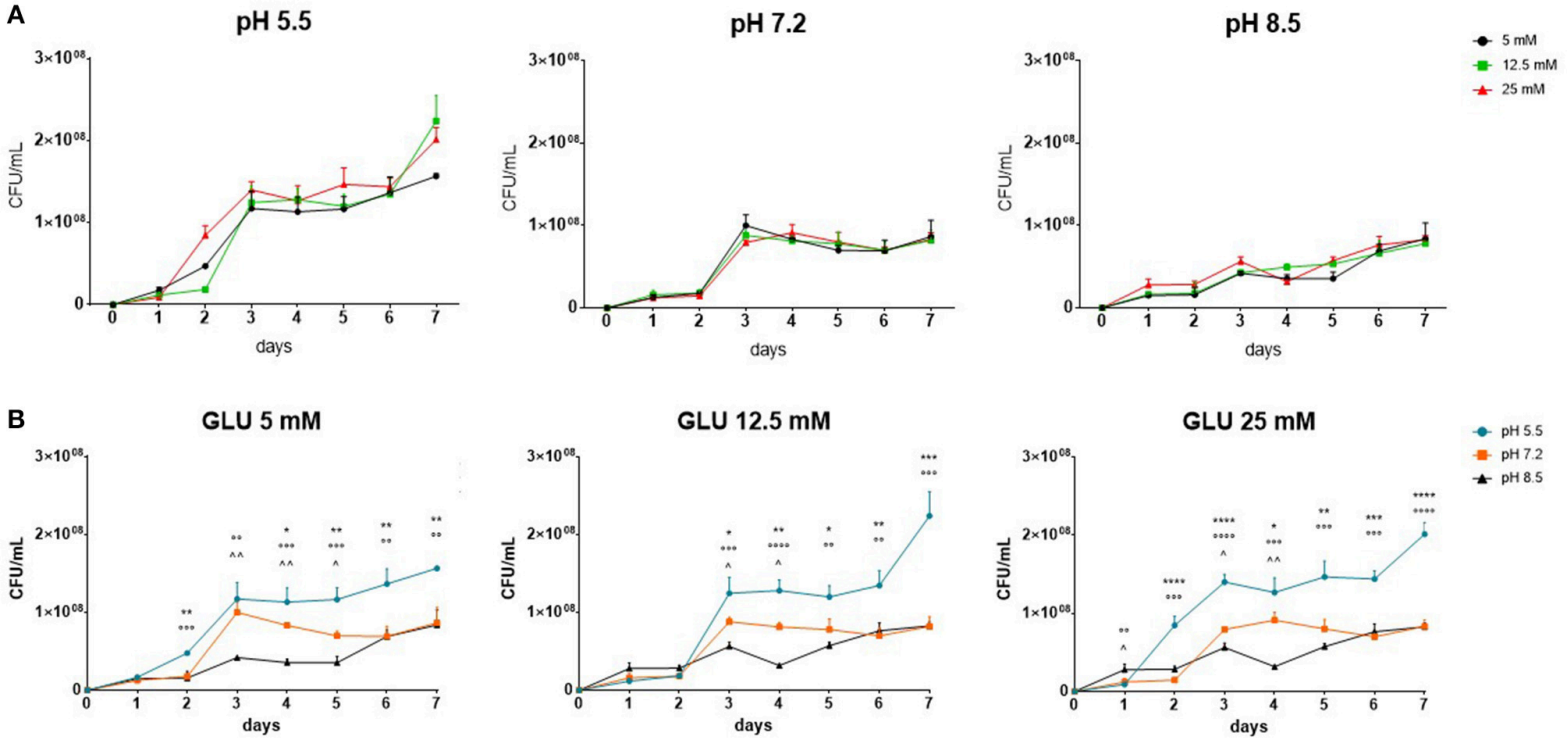
Kinetics of biofilm formation by *Myroides odoratimimus* in collagen gel matrix. Biofilm viability was assessed, by viable cell count, in SWF at different pH values (5.5, 7.2 and 8.5) and glucose concentrations (5, 12.5 and 25 mM) during a 7 day-incubation at 37°C. Results are shown as mean values + SDs (*n* = 6). Regression linear analysis showed a significant positive trend, regardless of pH and glucose concentration considered. Kinetics are stratified on **(A)** pH value or **(B)** glucose concentration. The number of symbols (^*^pH 5.5 vs. pH 7.2; °pH 5.5 vs. pH 8.5; ^∧^pH 7.2 vs. pH 8) indicates significance levels at ANOVA + Tukey's multiple comparison post-test: 1 (*p* < 0.05), 2 (*p* < 0.01), 3 (*p* < 0.001), and 4 (*p* < 0.0001).

Kinetics of biofilm formation in SWF was monitored throughout 7 days, at different pH values (5.5, 7.2, and 8.5) and glucose concentrations (5, 12.5, and 25 mM), and results are shown in Figure 2.

The linear regression analysis showed that biofilm's cellularity increased over time, regardless of pH values and glucose concentrations considered. However, at the same pH value no significant differences were observed among glucose concentrations (Figure 2A), whereas at the same glucose concentration the cellularity of *M. odoratimimus* biofilm significantly changed depending on pH value considered (Figure 2B). In general, at each glucose concentration tested biofilm growth was generally increased at pH 5.5, followed by pH 7.2 and 8.5.

### Antibacterial activity against planktonic and biofilm cells

**Figure 3:**
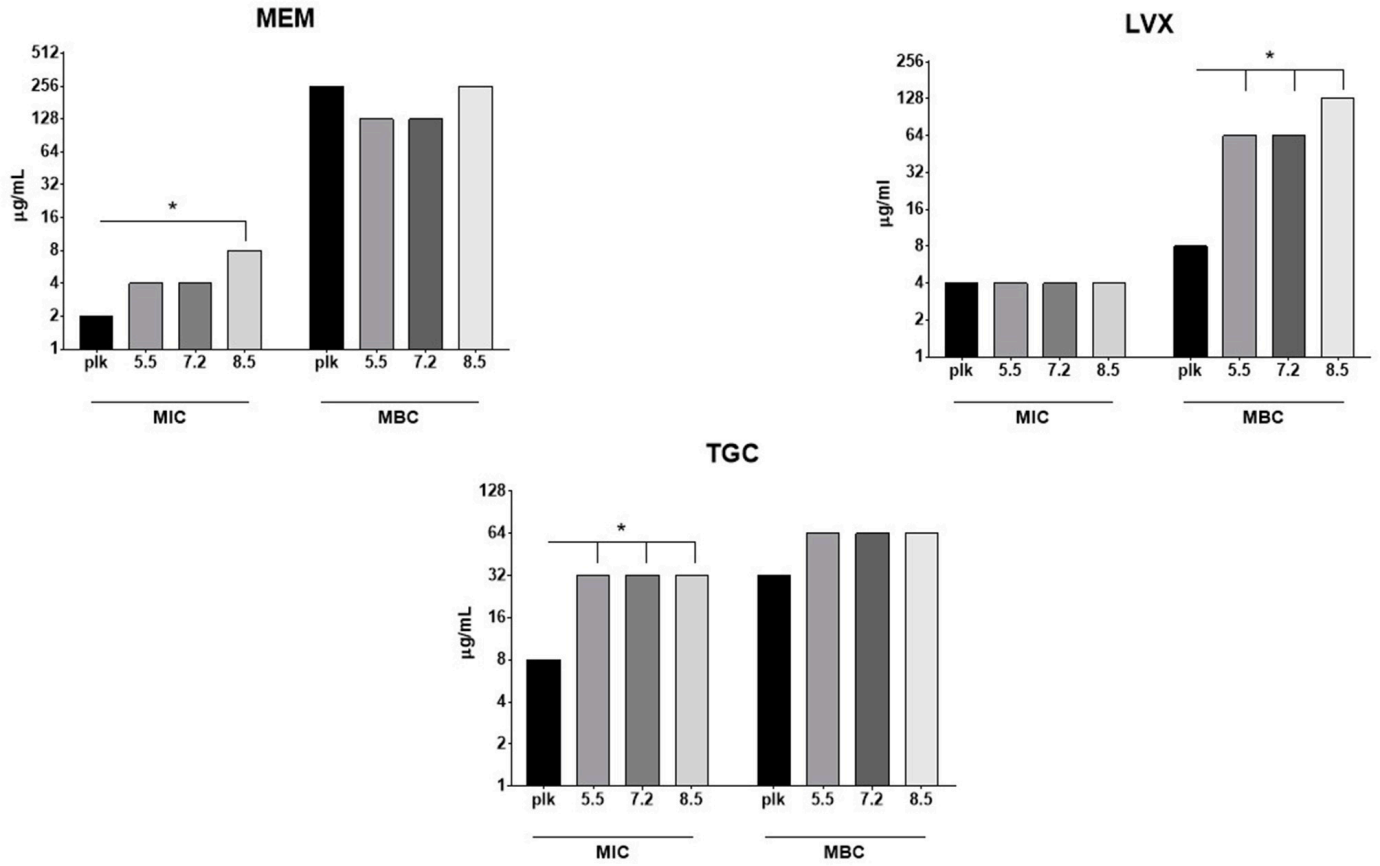
Antibiotic susceptibility of planktonic and biofilm *Myroides odoratimimus* cells. MIC and MBC values of several antibiotics were measured against planktonic (plk) and biofilm *Myroides odoratimimus* cells. Sessile (biofilm) cells were obtained following disruption of a 7 day-old biofilm grown in collagen gel matrix, in the presence of glucose 12.5 mM and at different pH values (5.5, 7.2, and 8.5) (bars with different scale of grays). ^*^Differences between MIC or MBC values were statistically significant if ≥2 log_2_. MEM, meropenem; LVX, levofloxacin; TGC, tigecycline.

MIC and MBC values of levofloxacin, meropenem, and tigecycline against planktonic and sessile *M. odoratimimus* cells are shown in Figure 3.

Meropenem and levofloxacin were the most active drugs against planktonic cells (MIC: 2 and 4 μg/ml, respectively), whereas tigecycline resulted to be the last one (MIC: 8 μg/ml). The comparative evaluation of MIC and MBC values by Killing Quotient (KQ = MBC/MIC) calculation (Cutler et al., 1994), showed that levofloxacin and tigecycline act by a bactericidal mechanism of action (KQ: 2 and 4, respectively), contrarily to meropenem (KQ: 128).

To evaluate if antibiotic susceptibility was dependent on biofilm mode of growth, the activity of each antibiotic was also tested against cells obtained following disruption of a 7 day-old biofilm grown in collagen gel matrix, in the presence of glucose 12.5 mM and at different pH values (5.5, 7.2 and 8.5). Our results showed that the antibiotic activity is not dependent on pH value, indicating levofloxacin and meropenem to be the most active drugs (MIC: 4 and 4/8 μg/ml, respectively). KQ values indicated that levofloxacin and meropenem are bacteriostatic (KQ: 32 and 16/32, respectively), whereas tigecycline is bactericidal (KQ: 2).

The comparative “planktonic-to-biofilm” analysis of MIC values showed that meropenem and tigecycline are significantly more active against planktonic compared to biofilm cells (ΔMIC ≥ 2 log_2_), whereas at pH 8.5 only for meropenem and regardless of pH in the case of tigecycline.

On the contrary, MBC values indicated levofloxacin being more active against planktonic cells compared to sessile counterpart (ΔMBC ≥ 2 log_2_), regardless of pH considered.

### Antibacterial activity against preformed biofilm

**Figure 4:**
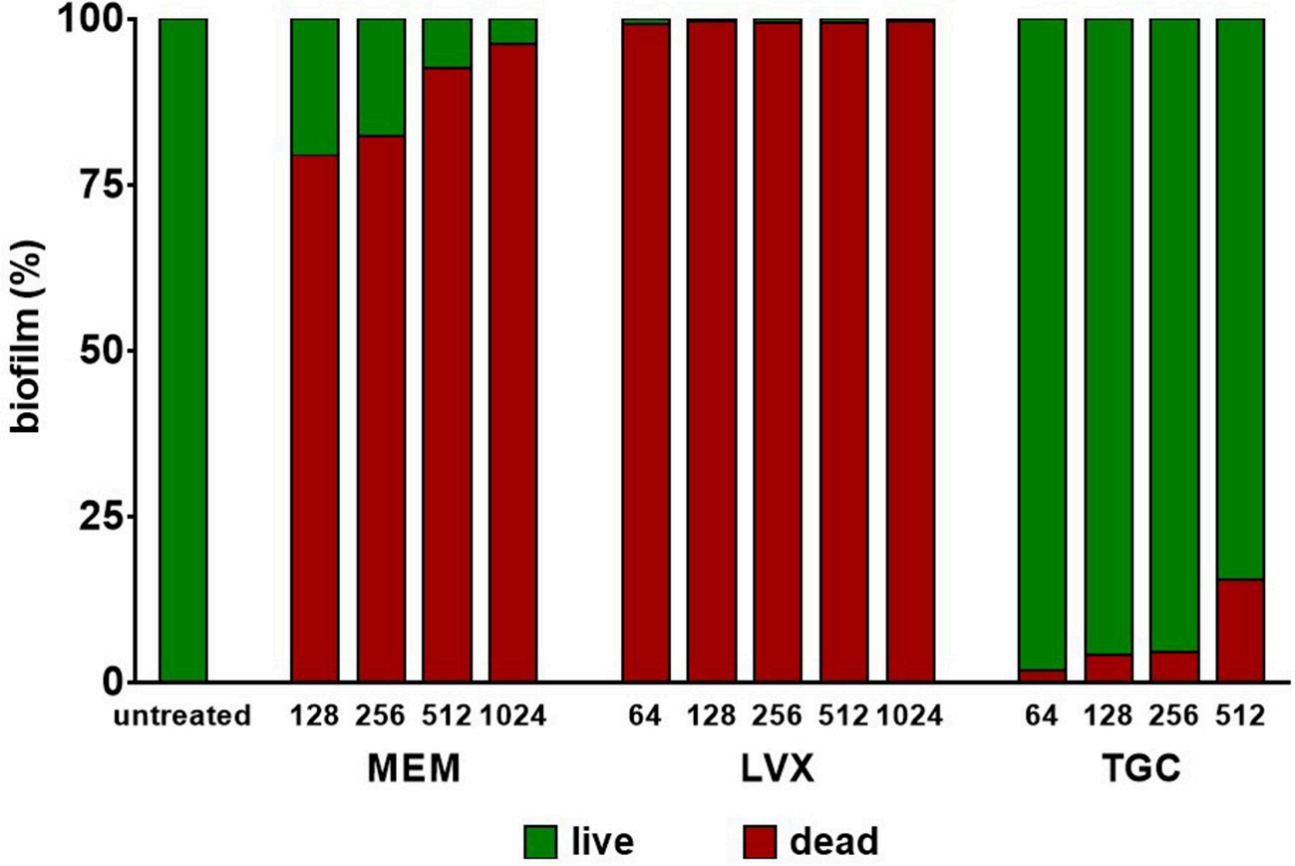
Activity of antibiotics against preformed biofilm by *Myroides odoratimimus* in collagen gel matrix. Biofilm was allowed to grow in collagen gel matrix following 7 day-incubation at 37°C in SWF medium at pH 7.2 and in the presence of glucose 12.5 mM. Biofilm was then exposed for 24 h to several concentrations of meropenem (MEM: 128–1,024 μg/ml), levofloxacin (LVX: 64–1,024 μg/ml) or tigecycline (TGC: 64–512 μg/ml). Biofilm's viability was evaluated by viable cell count and expressed as percentage compared to control (untreated) samples. The proportion of dead cells is shown in red, whereas that of live cells in green.

Preformed biofilm—allowed to grow, in collagen gel matrix, following 7 day-incubation at 37°C in the presence of SWF at pH 7.2 and glucose 12.5 mM—was challenged for 24 h with several concentrations of each antibiotic, and results are shown in Figure 4. None of the antibiotics tested were able to eradicate *M. odoratimimus* biofilm. However, exposure to meropenem and levofloxacin caused a significant reduction in biofilm's viability compared to control, regardless of concentration tested. The exposure to meropenem and levofloxacin caused a significant killing of preformed biofilm compared to the untreated control (*p* < 0.0001), ranging from 79.4% to 96.2% for meropenem, and from 99.1% to 99.6% for levofloxacin, depending on the concentration tested. Contrarily, exposure to tigecycline did not cause a significant reduction in biofilm's viability, regardless of concentration tested.

### Ultrastructural analysis of biofilm: CLSM

**Figure 5:**
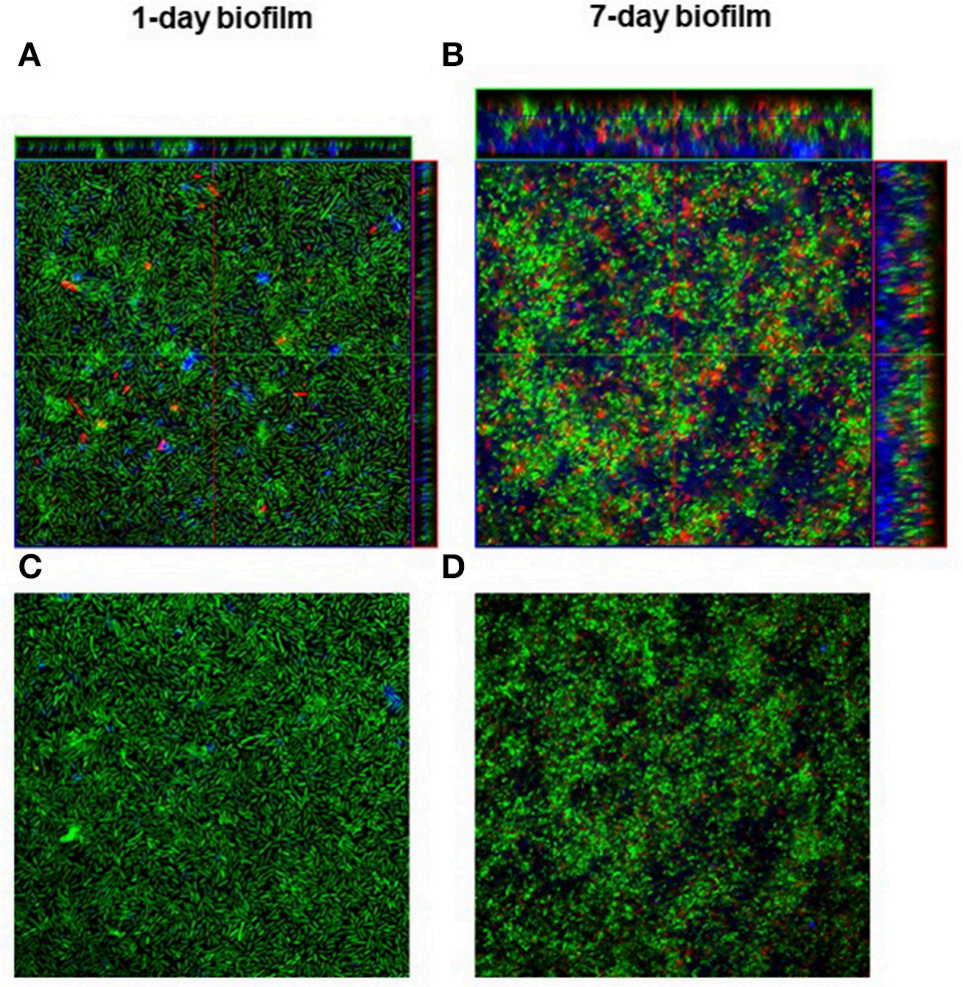
Confocal laser scanning microscopy of *Myroides odoratimimus* biofilm. Biofilm was allowed to grow, for 1 and 7 days, in collagen gel matrix in SWF (pH 7.2 and glucose 12.5 mM), and stained with BacLight Live/Dead kit. Green fluorescence: live cells; red fluorescence: dead cells; blue fluorescence: extracellular polymeric substance. **(A,B)** “multi-stack” reconstruction of the sample, with lateral x-y projections. **(C,D)** planar images.

Overall, CLSM analysis confirmed results from kinetics of biofilm formation by viable cell count, indicating a significant increase in cellularity over time (Figure 5).

Following 1 day of incubation (37°C, pH 7.2, glucose 12.5 mM), biofilm evenly formed showing a simple organization, mostly monolayer, as shown by lateral projections. On day 7 the biofilm structure increased in complexity, consisting of cellular “mushrooms” that significantly improved both thickness and structural heterogeneity. Furthermore, a large quantity of EPS appeared in the deeper layers of cellular community, as shown by ConA stain.

**Figure 6:**
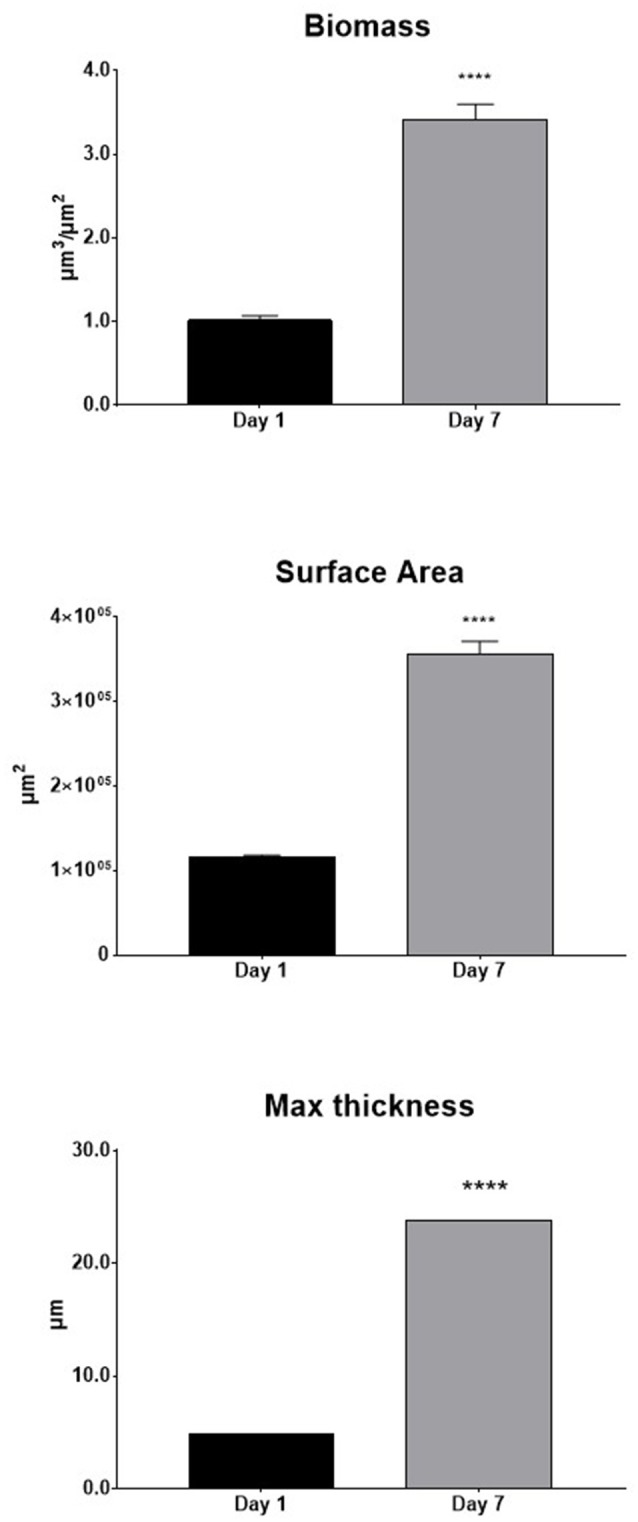
Structural organization of *Myroides odoratimimus* biofilm analyzed by COMSTAT computer program. Biofilm was allowed to grow in collagen gel matrix in SWF (pH 7.2 and glucose 12.5 mM) for 1 and 7 days, and then image stacks of biofilm were recorded by confocal laser scanning microscopy. Total biomass, surface area, and maximum thickness of biofilm samples were then quantitatively evaluated by COMSTAT software. Results are shown as mean values + SDs. ^****^p < 0.0001, paired *t*-test.

The structural organization of biofilm was analyzed by COMSTAT computer program, by evaluating three features for quantifying three-dimensional biofilm image stacks. Image analysis performed by COMSTAT confirmed that the structural complexity of biofilm significantly (*p* < 0.0001) increased from day 1 through day 7 in terms of total biomass, covered surface area, and maximum thickness (Figure 6).

### Ultrastructural analysis of biofilm treated with antibiotics at bactericidal concentration: confocal and electron microscopy

**Figure 7:**
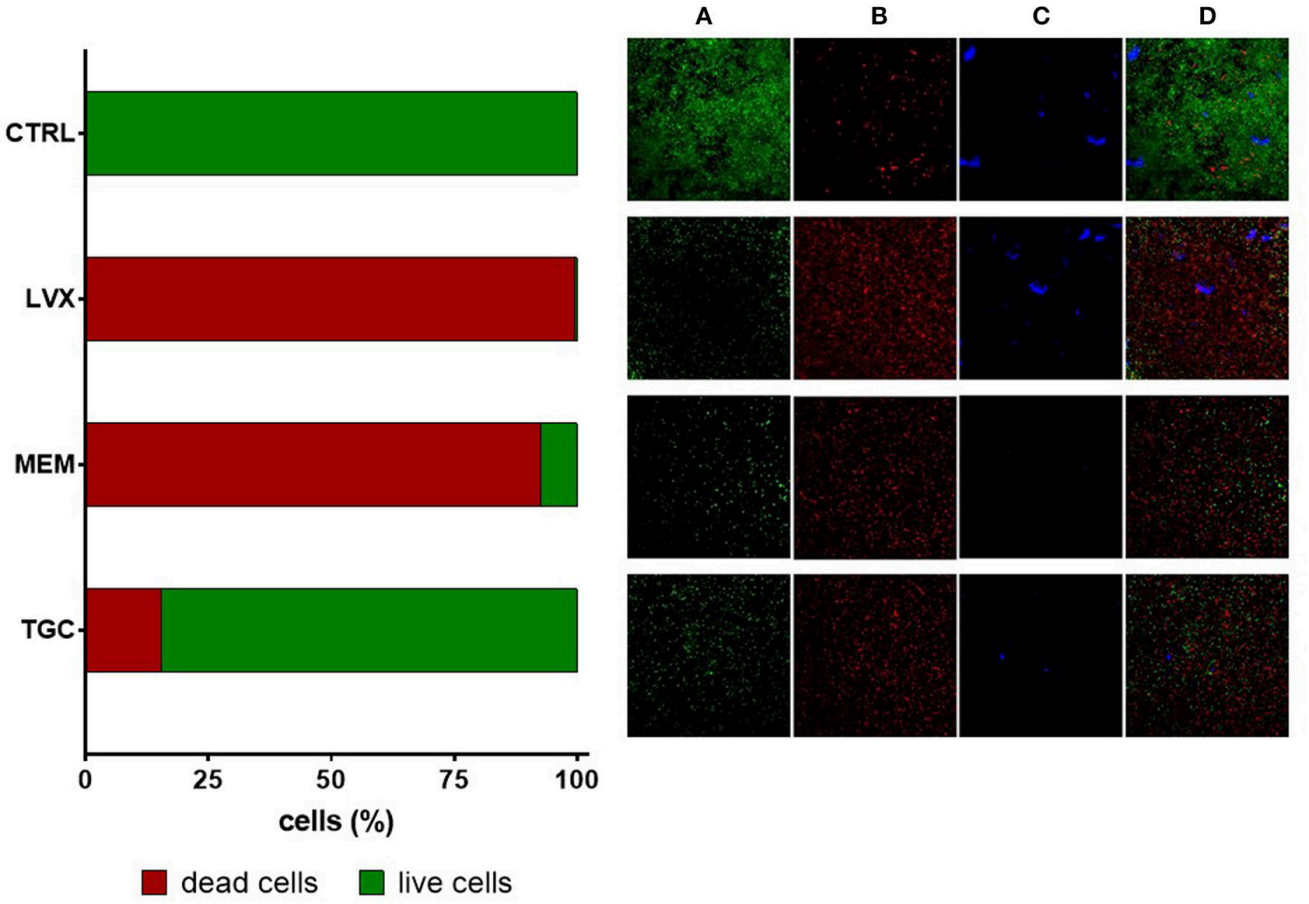
Confocal laser microscopy observation of preformed *Myroides odoratimimus* biofilm exposed to antibiotics at bactericidal concentration. Biofilm formed in collagen gel matrix (in SWF, pH 7.2, glucose 12.5 mM) for 7 days were exposed for further 24 h to levofloxacin (LVX), meropenem (MEM), and tigecycline (TGC) at 512 μg/ml. Control (CTRL) samples were exposed to SWF only. To the left: biofilm's viability was evaluated by viable cell count and expressed as percentage compared to CTRL samples. The proportion of dead cells is shown in red, whereas that of live cells in green. To the right: biofilms were stained with BacLight Live/Dead kit: **(A)** green fluorescence (live cells); **(B)** red fluorescence (dead cells); and **(C)** blue fluorescence (extracellular polymeric substance). **(D)** Co-localization of green, red, and blue fluorescence.

**Figure 8:**
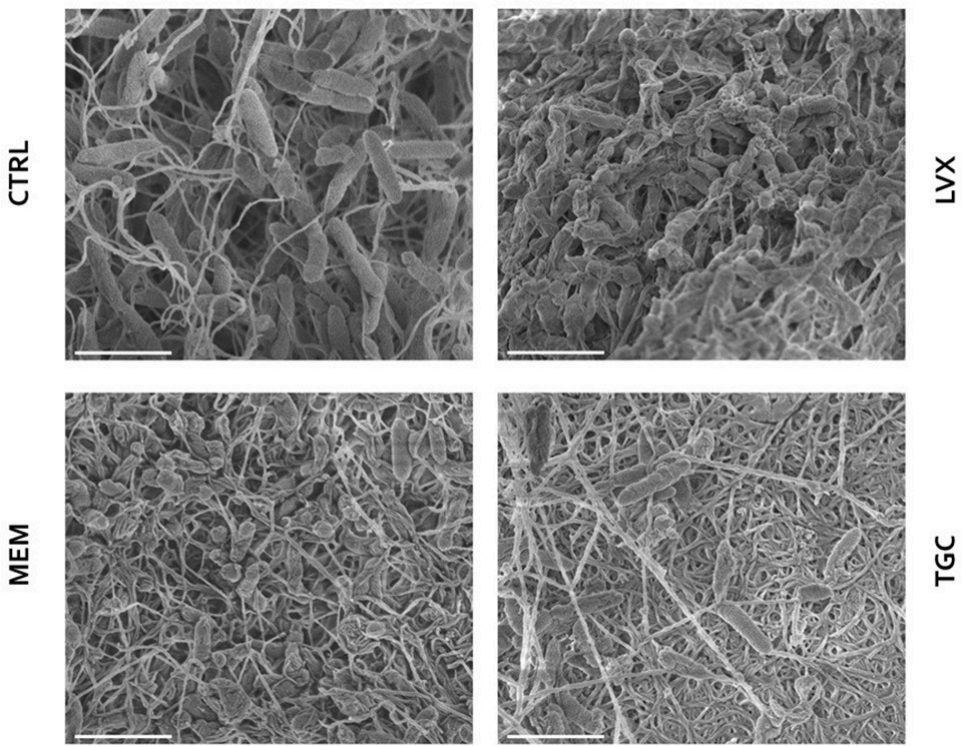
Scanning electron microscopy observation of preformed *Myroides odoratimimus* biofilm exposed to antibiotics at bactericidal concentration. Biofilm formed in collagen gel matrix (in SWF, pH 7.2, glucose 12.5 mM) for 7 days were exposed for further 24 h to levofloxacin (LVX), meropenem (MEM), and tigecycline (TGC) at 512 μg/ml. Control (CTRL) samples were exposed to SWF only. Scale bar: 2 μm.

**Figure 9:**
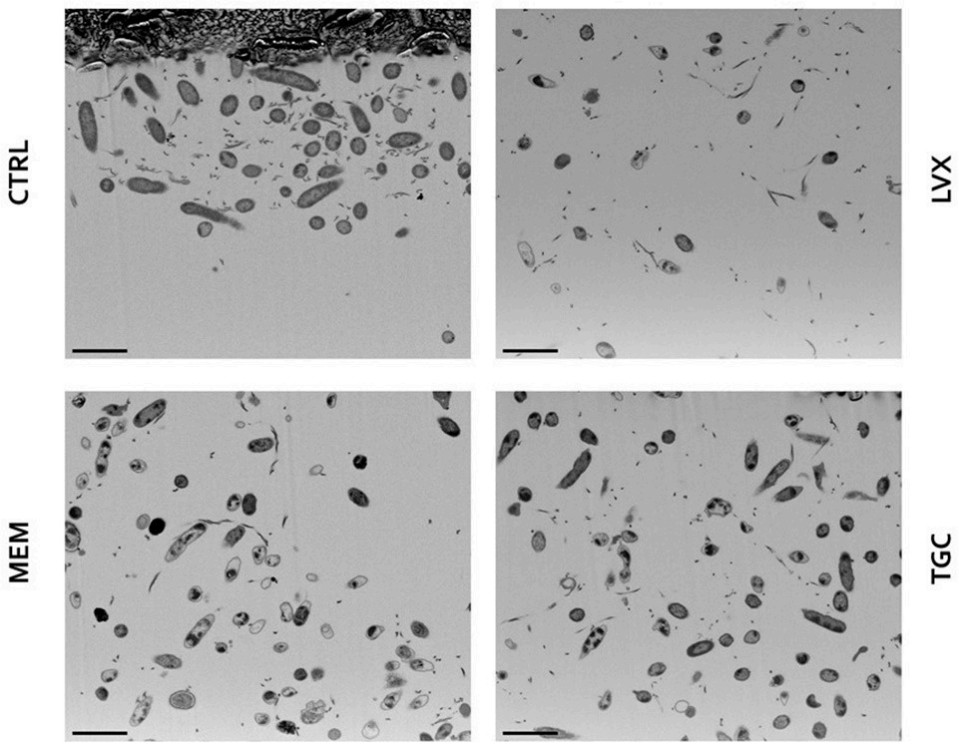
Focused-Ion-Beam Scanning Electron Microscopy observation of preformed *Myroides odoratimimus* biofilm exposed to antibiotics at bactericidal concentration. Biofilm formed in collagen gel matrix (in SWF, pH 7.2, glucose 12.5 mM) for 7 days were exposed for further 24 h to levofloxacin (LVX), meropenem (MEM), and tigecycline (TGC) at 512 μg/ml. Control (CTRL) samples were challenged with SWF only. Scale bar: 2 μm.

Preformed biofilms treated with levofloxacin, meropenem, and tigecycline at bactericidal 512 μg/ml concentration were observed both by CLSM (Figure 7) and FIB-SEM (Figures 8, 9).

In agreement with viable cell count results, CLSM showed that antibiotic exposure caused a significant reduction of biofilm's viability, although with striking differences among drugs. In particular, levofloxacin confirmed to be the most active molecule against mature biofilm, causing killing of most bacterial cells, followed by meropenem and the least active tigecycline (Figure 7).

SEM analysis highlighted that unexposed *M. odoratimimus* biofilm cells are interposed and partially attached to a loose and relaxed matrix, whereas antibiotic exposure caused cell structural damages and alteration to the matrix (Figure 8). Levofloxacin appeared as the most effective drug having 66% of the cells collapsed and shrank, followed by meropenem (33% of the cells), whereas tigecycline provoked minor changes in 14% of the cells. Biofilms treated with levofloxacin and meropenem also showed a compressed and shrank matrix composed of distinct filaments very close to each other, while tigecycline caused a reduction in matrix amount made of fused filaments.

The ultrastructural analysis performed by FIB/SEM showed that the most common morphological alterations caused by levofloxacin and meropenem is wall detachment from the membrane, while tigecycline provoked an irregularly indented wall with different degrees of deformity (Figure 9). In every treatment, some of the cells appeared empty. The matrix, appearing as a group of electron-dense filaments, was abundant in the control cells and significantly reduced in the treated cells.

### Gene expression in planktonic and biofilm cells

**Figure 10:**
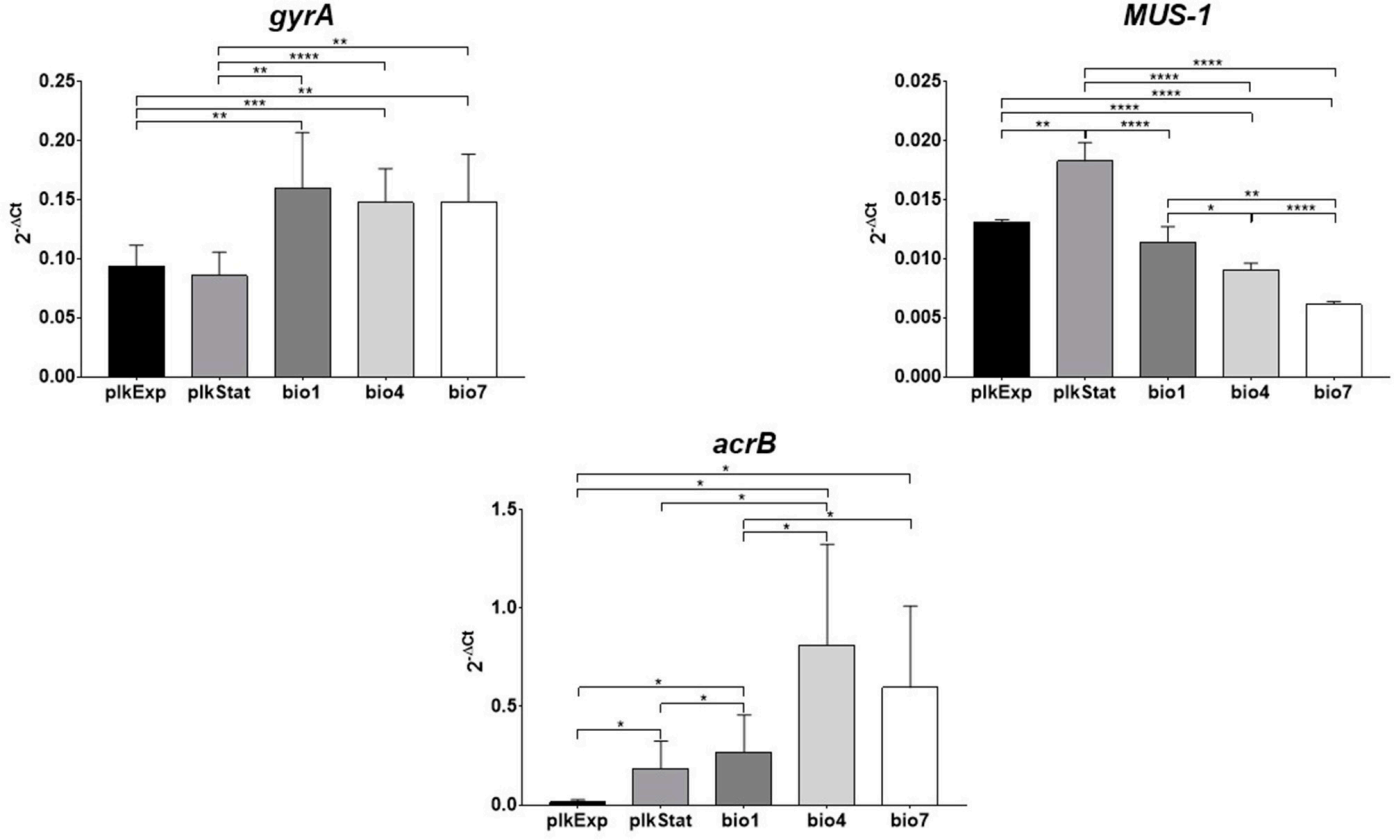
Assessment of *gyrA, MUS-1*, and *acrB* expression during planktonic and biofilm growth phenotypes of *Myroides odoratimimus*. RNA was isolated from both planktonic (plkExp: during exponential growth phase; plkStat: during stationary growth phase) and biofilm cells grown for 1 (bio1), 4 (bio4), and 7 (bio7) days in collagen gel matrix (37°C, pH 7.2, glucose 12.5 mM). The relative expression of each gene was then measured by RT-PCR and normalized to that of the housekeeping gene, according to the 2^−Δ*CT*^ method. Results are shown as mean + SD (*n* = 6). ^*^p < 0.05, ^**^p < 0.01, ^***^p < 0.001, and ^****^p < 0.0001, unpaired-t test.

Variations in *gyrA, MUS-1*, and *acrB* expression levels exhibited by *M. odoratimimus* under both planktonic and biofilm growth phenotypes are shown in Figure 10.

In planktonic cells, *MUS-1* and *acrB* expression levels were significantly higher during stationary growth phase compared to log-phase (at least *p* < 0.05), whereas no differences were observed for *gyrA*.

In biofilm cells, three different trends were observed, depending on gene considered. Particularly: (i) *MUS-1* expression significantly decreased over time, in a time-dependent manner (bio1 > bio4 > bio7; at least *p* < 0.05); (ii) an opposite trend was observed for *acrB*, whose expression increased in biofilm cells from day 1 throughout day 7 (bio1 < bio4 and bio7; *p* < 0.05); whereas iii) the expression of *gyrA* did not change over time.

During planktonic-to-biofilm lifestyle transition *gyrA* and *acrB* were significantly (at least *p* < 0.01) hyperexpressed, regardless planktonic growth phase and biofilm age, whereas an opposite trend was observed for *MUS-1* expression levels (*p* < 0.0001).

## Discussion

It has been recently observed that most of the bacteria causing chronic infections grow as biofilms, sessile communities that due to their structure and metabolic quiescence are inherently resistant to the action of numerous antibiotics as well as the immune response of the host (Percival et al., 2012; Rahim et al., 2017). Several experimental and clinical evidences reported in the literature indicated the presence *in situ* of microbial biofilms in chronic wounds, thus contributing to a delayed healing (Gristina et al., 1985; James et al., 2008; Kirketerp-Møller et al., 2008; Bjarnsholt et al., 2010; Kennedy et al., 2010; Fazli et al., 2011; Han et al., 2011; Neut et al., 2011).

Based on these evidences and the recurring nature of the ulcer observed in the case described above, we have hypothesized that the strain we isolated could be able to grow in the form of biofilm. Our hypothesis is also supported by a number of evidences from the literature suggesting the existence of a relationship between infection and the presence of prosthetic implants, ideal substrates for the formation of bacterial biofilms. In particular, Jover-Sáenz et al. (2016) described a case of *M. odoratimimus* infection of a knee prosthesis. Likewise, during an outbreak recorded in a Turkish hospital, most *M. odoratimimus* strains had been isolated from the urine of long-acting urinary catheter patients (Yagci et al., 2000). To these evidences, we also add direct evidence of the ability to form multi-species biofilms by *M. odoratus* isolated from food samples and tilapia (Bremer and Monk, 2001; Jacobs and Chenia, 2009).

To verify our hypothesis, we first evaluated the ability of *M. odoratimimus* to form biofilm on polystyrene by crystal violet assay. The results indicated that this microorganism can be classified as a “strong biofilm-producer” (Stepanović et al., 2007) being able to produce a significant amount of biofilm biomass and that this ability significantly increases over time (Figures 1A–D), therefore suggesting a possible role of biofilm formation in the chronicization of the calcaneal infection.

Starting from these preliminary experimental evidences, in the second step of this work we evaluated biofilm formation and its susceptibility to antibiotics under conditions relevant to the case report we described (Pompilio et al., 2017).

To this end, we used an experimental model to simulate the “wound environment” (Figures 1E,F; Werthén et al., 2010). The main features of the model are the presence of a highly viscous 3D-matrix containing I type collagen and of a “Simulated Wound Fluid” (SWF). Type I collagen, a fibrous extracellular matrix protein, is the main component of the skin dermis and, properly polymerized, it forms a gelatinous and slightly cross-linked matrix that makes it suitable to simulate a “skin-like” environment. SWF consists of equal parts of bovine fetal serum and 0.1% peptone water, to simulate the composition of the serous exudate. Using this model, we observed that bacteria are organized, in the thickness of the collagen matrix, in communities showing structural and organizational characteristics (Figure 5) of biofilms not directly adhered to a surface, similar to those recently observed in chronically infected wound tissue (Kirketerp-Møller et al., 2008; Bjarnsholt et al., 2009).

Skin pH is one of the most important factors for healing wounds (Kaufman and Berger, 1988) since processes related to cell biosynthesis, metabolism transport and cell cycle progression are dependent on both intracellular and extracellular pH (Wahl et al., 2002). There is a precise relationship between the changes in the topical pH and the various healing phases (Schneider et al., 2007). It has been shown that acidic pH improves wound healing, while pH values suggestive for an alkaline environment (7.1 < pH < 8.9) favor wound chronicization (Wilson et al., 1979; Hoffman et al., 1999; Gethin, 2007)^1^ The fall in pH, due to hypoxia (Leveen et al., 1973) and increased lactic acid production (Hunt et al., 1967), favors in fact healing improving the proliferation and migration of fibroblasts and regulating bacterial colonization. Contrarily, in the presence of alkaline pH bacterial proliferation and alteration of extracellular matrix molecule synthesis is favored therefore leading to the chronicization of infection (Thomas et al., 1993; Schneider et al., 2007). Exposure of the underlying tissues to alkaline pH values is also a viable route of resident skin flora that can colonize the wound that becomes chronic.

It is well known in the literature that glucose can modulate biofilm formation. In *S. aureus* and *Staphylococcus epidermidis* it promotes the formation of biofilms inducing over-expression of the genes organized in the *icaADBC* operon which encodes the enzymes required for the synthesis of the polysaccharide intercellular adhesin (PIA), indispensable for the formation of microcolonies, the first biofilm units (Götz, 2002). Contrarily, in *Escherichia coli* glucose inhibits the thickening of a biofilm already formed but is not capable of causing its disruption, suggesting that it interferes with cell adhesion to biofilm (Jackson et al., 2002). In addition, the strain used in the present study was isolated from a diabetic patient, therefore we have found interesting to evaluate the effects on *M. odoratimimus* biofilm formation of glucose at concentrations relevant to different stages of diabetic disease (American Diabetes Association, 2017).

To date no studies have specifically looked at wound-derived bacteria and the effect of pH and glucose on their growth and formation of a biofilm within a wound. Therefore, we used the “skin-like” model to evaluate the effect of different pH values and glucose concentrations on *M. odoratimimus* biofilm formation kinetics over a 7-day period: (i) pH: 5.5 (intact skin, and stage I ulcer), 7.2 (stage II ulcer), and 8.5 (chronic ulcer, and stage III ulcer); (ii) glucose concentration: 5 mM (normoglycemic patient), 12.5 mM (diabetic patient), and 25 mM (severe diabetic patient).

Linear regression analysis indicated that biofilm's cellularity significantly increases over time (Figure 2), regardless of pH value or glucose concentration considered. However, variations in glucose concentration had no effect on biofilm formation, regardless of the pH values considered (Figure 2A). Conversely, as pH changed significant variations in biofilm's cellularity were observed. In general, biofilm grown at pH 5.5 showed higher cellularity than that obtained at other pH values (Figure 2B). In disagreement with our findings, Hostacká et al. (2010) observed that biofilm amount formed by *P. aeruginosa, Klebsiella spp*., and *Vibrio cholerae* increases as pH increased (pH 7.5 and 8.5 > pH 5.5). Such discrepancy might be due to crystal violet assay they used for quantitative biofilm analysis. In fact, it evaluates not only the cellular component of a biofilm but also EPS, although it does not provide any information on cell viability. Higher biofilm levels found by Hostacká et al. (2010) might be therefore due to an increased EPS production and/or to stained dead, but not detached, cells. Our results indicated a virulence trait of *M. odoratimimus* that is the ability to grow as biofilm at pH values that, on the intact skin, generally regulate the proliferation of the endogenous commensal bacterial flora, thus preventing the emergence of infectious processes (Sharpe et al., 2009).

Observation by CLSM (Figure 5) confirmed the results from biofilm formation kinetics monitored by viable cell count, indicating that structural complexity of *M. odoratimimus* biofilm increases over time. We observed, in fact, a transition from a simple monolayer, following 1 day of incubation, to a much more complex organization after 7 days of incubation, with features of a classic “mature” biofilm where the alternation of void spaces and “mushrooms” significantly increased both structural heterogeneity and thickness. In addition, CLSM analysis confirmed that maturation of *M. odoratimimus* biofilm leads to increased cellularity and EPS amount that is mainly localized in the deeper layers of the sessile community. Image analysis by COMSTAT software confirmed the greater complexity of the mature 7 day-old biofilm, compared to that obtained after 1 day of incubation, regarding increased biomass, surface area covered, and maximum thickness (Figure 6).

The clinical relevance of a microbial biofilm lies mainly in its decreased sensitivity to antibiotic action compared to the planktonic counterpart (Flemming et al., 2016). The susceptibility of preformed biofilm by *M. odoratimimus* to tigecycline, meropenem and levofloxacin—selected because they were used for the treatment of the recurrent calcaneal infection we recently described Pompilio et al. (2017)—was then evaluated under experimental conditions relevant to the clinical case considered, that are: pH 7.2 (stage II ulcer), 12.5 mM glucose (chronic stage II diabetes, diabetic), and 7 days-incubation (chronic/recurrent infection).

To this end, preformed biofilms of *M. odoratimimus* were exposed for 24 h to scalar concentrations of each antibiotic, then biofilm viability was assessed by viable cell count. Overall, our results indicated that *M. odoratimimus* biofilm is significantly more resistant to all tested antibiotics, compared with the planktonic counterpart. None of them, in fact, was able to eradicate the biofilm, even when tested at maximum bactericidal concentrations (1.024 μg/ml, corresponding to 128x and 4xMBC, respectively for levofloxacin and meropenem; 512 μg/ml, corresponding to 16xMBC for tigecycline; Figure 4). However, striking differences in the efficiency against preformed biofilm were observed among antibiotics. In particular, exposure to meropenem or levofloxacin resulted in a highly significant reduction in biofilm vitality, with a concentration-independent effect. Conversely, tigecycline did not cause significant vitality reduction even after exposure to the highest concentration (512 μg/ml, equivalent to 16xMBC). The ability to deliver so high concentrations of antibiotic directly to the site of infection can be achieved only through topical administration, thus offering several advantages including ready access to the desired site, increased therapeutic efficacy (lower chance to develop antibiotic resistance, effective against resistant strains since concentration is well higher than MIC), decreased systemic exposure/toxicity, as well as the ability for individualized treatment options (i.e., multiple therapeutics simultaneously).

The observation at the scanning laser confocal microscope (Figure 7) and the electronic transmission microscope (Figures 8, 9) confirmed results from viable cell counts. In particular, electronic microscopy showed a greater activity of levofloxacin against preformed biofilm The percentage of non-viable cells or with impaired morphology was found to be greater following levofloxacin treatment (approximately 60%) compared to meropenem and tigecycline (33 and 14%, respectively). The cell lysis was secondary to cell wall damage, with strongly suggestive wall detachment (levofloxacin, meropenem) rather than more pronounced deformations and/or breaks (tigecycline). Both optical and electronic microscopy showed that antibiotic treatment caused a reduction of EPS, regardless of the molecule considered.

The inherent antibiotic-resistance showed by a microbial biofilm can be the result of a “resistance” or “tolerance.” Resistance is of genetic origin, being acquired both by mutations and by gene transfer, and therefore it is proper to the sessile cells also following biofilm's dispersion (Flemming et al., 2016). By contrast, the term “tolerance” refers to a characteristic that is specific to the biofilm growth phenotype (Olsen, 2015; Brauner et al., 2016), and consequently is lost secondarily to the dispersion of cells (Königs et al., 2015; Thuptimdang et al., 2015). Tolerance can be the result of both the biofilm EPS properties—which acts as a “barrier” by sequestering or inactivating the antibiotic—and the slow microbial growth secondary to a quiescent metabolism (Flemming et al., 2016).

To define the real mechanism underlying reduced susceptibility to antibiotics by *M. odoratimimus* biofilm, MBC values against planktonic cells and those (sessile) harvested following biofilm's disruption were measured and comparatively evaluated. The activity of levofloxacin was significantly greater against planktonic cells than sessile counterparts (ΔMBC ≥ 2 log_2_), regardless of the pH value considered (Figure 3), therefore indicating a genetically acquired resistance. Conversely, the unchanged susceptibility to meropenem and tigecycline might be attributed to a structural feature of the biofilm and must therefore be defined as “tolerance”. Interestingly, meropenem, known to be generally bactericidal, showed bacteriostatic activity, whereas tigecycline, generally known as bacteriostatic, was found to be bactericidal against *M. odoratimimus*. Further studies are needed to clarify these apparently unusual findings.

To further unravel the mechanisms underlying intrinsic resistance of biofilm, changes in the expression of three genetic determinants (*gyrA, MUS-1, acrB*) involved in *M. odoratimimus* antibiotic resistance (Hu et al., 2017) were monitored by RT-PCR both in planktonic and biofilm cells. Our results indicated that during the transition from planktonic to biofilm growth phase the expression of both *acrB* and *gyrA* significantly increases (Figure 10), whereas that of *MUS-1*, codifying for a chromosomally encoded β-lactamase, did not significantly change. These findings might explain the inherent antibiotic resistance of *M. odoratimimus* biofilm to tigecycline, since *acrB* codifies for the AcrB subunit of the active RND-type AcrAB-TolC efflux pump, able to excrete against concentration gradient different classes of antibiotics, such as tigecycline (Sun et al., 2014). The level of resistance probably increases during biofilm aging as suggested by the direct relationship observed in sessile cells between expression levels and time (Figure 10). In agreement with our findings, several studies have previously demonstrated an association between elevated tigecycline MICs and overexpression of *acrAB* in *Enterobacter cloacae* (Keeney et al., 2007) and *Proteus mirabilis* (Visalli et al., 2003), although in planktonic cells. On the other hand, the increased *gyrA* expression might explain why *M. odoratimimus* biofilm are more susceptible to levofloxacin.

In conclusion, overall our results clearly indicated that *M. odoratimimus*, under conditions relevant to a wound site in a diabetic patient, can form quantitatively and qualitatively complex biofilms. This feature could play an important role in the chronicization of wound infections due to the intrinsic resistance of biofilm to antibiotics. In this regard, our results showed that both levofloxacin and meropenem could represent “first choice” molecules for the treatment of biofilm-related infections caused by *M. odoratimimus*. These findings highlight to reconsider the etiological significance of *M. odoratimimus* isolation and its pathogenicity, not only in the immunocompromised patient. Further *in vivo* and *in vitro* studies are warranted to confirm the clinical significance of our results.

## Author contributions

AP and GD designed and drafted the work. AP, GG, FV, MM, and AD provided substantial contributions to the acquisition and analysis of data for the work. AP, GD, FV, and AD revised the work critically for important intellectual content.

### Conflict of interest statement

The authors declare that the research was conducted in the absence of any commercial or financial relationships that could be construed as a potential conflict of interest. The reviewer MR and handling Editor declared their shared affiliation.

## Footnotes

^1^*Physical Changes in Dermal Tissues Around Chronic Venous-Technische Informationsbibliothek (TIB)*. Available online at: https://www.tib.eu/de/suchen/id/BLCP%3ACN032658576/Physical-changes-in-dermal-tissues-around-chronic/.
